# Structure and process of palliative care provision: a nationwide study of public hospitals in Thailand

**DOI:** 10.1186/s12913-021-06623-w

**Published:** 2021-06-29

**Authors:** Parichat Dokmai, Natthani Meemon, Seung Chun Paek, Supakarn Tayjasanant

**Affiliations:** 1 Department of Society and Health, Faculty of Social Sciences and Humanities, Mahidol University, Salaya, Phutthamonthon, Nakhon Pathom 73170 Thailand; 2 Siriraj Palliative Care Center, Faculty of Medicine Siriraj Hospital, Mahidol University, Bangkoknoi, Bangkok, 10700 Thailand

**Keywords:** Palliative care, End-of-life care, Donabedian, Facility-based care, Structural equation model, Thailand

## Abstract

**Background:**

The demand for palliative care in hospitals in Thailand has rapidly increased in recent years. Subsequently, the way in which palliative care systems should be arranged to facilitate the care process and patient preparation for their end stage of life is still an ongoing debate among policy makers and researchers. Although palliative care is provided in most facilities, there is no clear protocol for palliative care due to a lack of empirical evidence. Thus, this study attempts to analyse the situation and quality of palliative care provision in Thai public hospitals.

**Methods:**

A cross-sectional study was conducted in 2018. A questionnaire with measures concerning hospital characteristics, the structure of palliative care provision, and processes related to achieving a good death was developed. The questionnaire was sent to all 862 public hospitals across 76 provinces, and the response rate was 62.88%. A structural equation model was specified to operationalize Donabedian’s framework. To our knowledge, this is the first nationwide study to investigate facility-level palliative care provision in Thailand.

**Results:**

The study results confirmed the relationships between the structure and process of palliative care provision in hospitals. The sufficiency and competency of doctors and nurses and the variety of relaxation equipment were either directly or indirectly associated with the process components relevant to the response to the patient’s needs, effective communication, and respect for the patient’s dignity. In addition, the performance of palliative care research in hospitals was associated with the response to the patient’s needs and effective communication, while the allocation of physical areas was associated with effective communication.

**Conclusion:**

This model can be used to evaluate the overall situation of palliative care provision at the national level. It could also contribute to the development of standard measurements for evidence-based palliative care quality improvement in hospitals.

## Introduction

The increasing demand for facility-based palliative care in Thailand has attracted the attention of healthcare professionals, scholars, and policy makers [1]. It has been reported that the number of patients needing palliative care in public hospitals increased almost five times from 13,399 in 2014 to 64,182 in 2019 [2]. This highlights the need to advance the country’s palliative care provision to ensure quality of care as well as the quality of life of the patients.

The development of palliative care in Thailand is in the preliminary stage of integration into mainstream health care services, as palliative care has reached a critical mass in terms of provision in most locations through regular support with respect to drugs, capacity and policy implementation [3]. However, many challenges regarding the structure of palliative care at the facility level have been reported. The challenges include inadequate palliative care training for medical and nursing staff, a lack of intra-facility coordination among specialists, the high workload of hospital staff, the unavailability of drugs in smaller facilities, a lack of medical equipment, the limited number of studies that could be used to promote quality of care and quality of life for patients, and a lack of protocols for nonmedical practices such as communication with patients and psychosocial-spiritual support [1, 4–8].

The way in which palliative care systems should be arranged to facilitate the care process and patients’ preparation for their end stage of life (i.e., good death) is still an ongoing debate. There have been continuing efforts to develop a protocol for palliative care provision in the Thai context. In fact, many guidelines and quality of care assessment tools are available for wider use [9–11]; nevertheless, most Thai hospitals report following the guidelines developed by each facility [12]. Despite the nationwide implementation of palliative care performance indicators related to some care process (e.g., opioid drug provision, advance care planning, home visits) [13, 14], there is no clear protocol for the provision of facility-based palliative care in Thailand [12]. To date, assessments of the status and quality of palliative care provision across medical facilities are still limited.

Herein, we use facility-level data that can typically be obtained from public hospitals that offer palliative care to investigate how hospitals have arranged palliative care provision systems and whether these arrangements could be linked to palliative care processes that enhance patient quality of life. This nationwide study, hence, is the first step in exploring the current situation of facility-based palliative care provision in Thailand. The evidence from this study offers meaningful insight for the development of common quality measurement tools and ultimately the improvement of palliative care quality in Thailand.

## Framework

The Donabedian quality of care framework has been widely used in prior work to confirm the links between the structure and process of palliative care [15–20]. Conceptually, quality of care measures comprise three categories: structure, process and outcome. Structure refers to organizational features or characteristics that are presumed to have an impact on the performance of health care organizations [21]. Structural challenges might be the cause of problems identified in the process of care. The palliative care process emphasizes the actions of healthcare providers and the interactions healthcare providers have with patients and family members. The presence of clear practice guidelines as well as the availability and quality of human and material resources could determine how well patients’ physical, psychosocial and spiritual needs are responded to and respected [22–25]. A good process of care reflects good preparation for patients’ end stage of life, which eventually leads to good palliative care outcomes [26, 27].

## Methods

### Participants and procedures

We approached hospital directors from all 862 public hospitals operating under the Ministry of Public Health across 76 provinces in Thailand. After obtaining informed consent, the director of each hospital assigned one medical staff member working in palliative care as a representative to provide information about palliative care in his/her respective facility. The respondents were given the option of completing the questionnaire online, with a hard copy, or by telephone interview. Where possible, a reminder was sent to respondents 2 to 4 weeks after they received the questionnaire. This study was approved by the Ethics Committee, Mahidol University Institutional Review Board and the participating hospitals that required the study to be approved by their own ethics committee (Certificate of Approval No.: 2017/185.0509) (MU-SSIRB No.: 2017/222 (B2)).

A total of 542 hospitals returned completed questionnaires, yielding a response rate of 62.88%. The respondents were registered nurses (55.0%), head nurses (43.7%) and hospital directors (1.3%). Most of them were female (97.6%). The average age was 44.4 years (SD = 8.21). The average number of years of work experience in palliative care was 4.04 (SD = 3.09). Regarding their classification by service capacity and size, the participating hospitals included 17 (3.1%) advanced-level tertiary care hospitals (400–1000 beds), which serve as referral centres at the regional level; 63 (11.7%) standard-level and middle-level tertiary care hospitals (150–500 beds), which serve as referral centres at the provincial level; and 462 (85.2%) first-level and middle-level secondary care hospitals (10–120 beds), which provide care at the district level. The distribution of participating hospitals was highly similar to that at the country level and that across each region in the country.

### Measures

#### Hospital characteristics

Data on the hospital *type* (level of service capacity) and *size* (number of beds) were collected and analysed to understand the characteristics of the participating hospitals.

#### Structure of palliative care

Structure in this study is defined as the hospital context in which palliative care is provided. This includes human resources and management, medical and nonmedical equipment, research activities and physical space.

Human resources include latent and manifest variables. *The sufficiency of medical personnel* is a latent variable comprising two observed indicators: the ratio of palliative care doctors to palliative care patients and the ratio of palliative care nurses to palliative care patients. *Staff competency* is represented by two manifest variables: the number of doctors and the number of nurses who had attended at least 1 month of palliative care training. *The availability of a multidisciplinary team* is measured as the number of medical and nonmedical specialist types on the hospital’s assigned palliative care team. The availability of nonmedical staff, who generally play important roles in palliative psychosocial care, is represented by two manifest variables: the *number of social workers* and the *number of psychologists in the hospital.*

The availability of medical and nonmedical equipment variables reflects the hospitals’ capacity to provide palliative care. We measured the *variety of medical equipment* and *variety of relaxation equipment* by counting the types of medical equipment necessary for palliative care that are available for palliative care patients, such as syringe drivers, infusion pumps, airbeds, and oxygen concentrators, as well as the types of relaxation equipment or physical space, such as media players, magazines, novels and religious practice rooms, available to patients. *The availability of medicine* is measured by the number of types of pain control drugs. *Research on palliative care* and *the presence of designated areas for palliative patients* were measured dichotomously to indicate whether the hospital has performed any research related to palliative care and whether there is space designated specifically for palliative care inpatients.

#### Process of palliative care

The structural components of palliative care are hypothesized to affect hospitals’ practices, leading to a good dying process. *Time spent by doctors* and *time spent by nurses* refer to the average amount of time (in minutes) palliative care doctors and nurses, respectively, spend in active conversation with each palliative care patient.

The process of preparing patients and/or their family caregivers for the end stage of life is classified into three concepts: response to physical and psychological needs, effective communication, and respect for patients’ needs/dignity. The latent variable *response to physical and psychological needs* has five indicators measured as the percentage of palliative patients receiving care according to the guidelines for different aspects of care: pain management, delirium management, depression management, spiritual care management and bereavement care management. *Effective communication* has four indicators measured as the percentage of patients who received bad news from trained medical staff, who received bad news in compliance with related guidelines, who received advice from trained advance care planning staff, and who received advice related to advance care planning guidelines. *Respect for patient* has three indicators: percentage of patients or patients’ family members who participated in decision-making about medical treatment, decision-making about place of death and the facilitation of making a written living will.

### Statistical analysis

To model simultaneous relationships among multiple latent constructs and manifest variables, we employed a structural equation modelling (SEM) approach. Confirmatory factor analysis (CFA) was performed to examine the validity of the measurement for each construct. Model fit assessment was conducted after performing model specification to evaluate how well a specified model fit the data. The threshold for acceptable goodness of fit is a comparative fit index (CFI) > .95, standardized root mean square residual (SRMR) < 0.08 and root mean square error of approximation (RMSEA) < 0.08 [28].

## Results

### Characteristics of the study sample

Across the 542 hospitals, the average number of hospital beds was 103.15 (SD = 147.12). In terms of the sufficiency of medical personnel, the average doctor-to-palliative patient and nurse-to-palliative patient ratios were 0.38 (SD = 0.42) and 1.61 (SD = 2.11), respectively. Only 56 hospitals (10.3%) reported having doctors and 274 hospitals (50.6%) reported having nurses who attended at least 1 month of palliative care training. Hospitals with higher capacity appeared to have a larger number of palliative-care-trained medical staff. Most hospitals reported that they had a multidisciplinary team for palliative care; these teams comprised an average of 5.13 (SD = 1.55) types of medical and nonmedical professionals (e.g., physicians, registered and practitioner nurses, nurse aids, pharmacists, social workers, psychologists, physical therapists, nutritionists, music therapists, Thai traditional massagers). Sixty-three (11.6%) and 182 (33.6%) hospitals reported having at least one social worker and at least one psychologist, respectively.

Pain medications that are generally used for palliative care patients, such as tramadol and morphine injections, were available in most hospitals (98.0 and 96.5%, respectively). Hospitals with higher capacity had a greater variety of pain medication. In terms of medical equipment, most hospitals had air mattresses, oxygen concentrators and infusion pumps (78.2, 68.1 and 63.8%, respectively), while 44.5% had syringe drivers. There was a variety of relaxation equipment available for palliative care patients. In addition to general media such as video, radio, TV and reading materials, most hospitals had Dhamma books and cassettes (89.5 and 72.7%, respectively), and some had Christian tabernacles (22.7%) and/or Islamic prayer rooms (11.1%). The average number of relaxation equipment types was 3.24 (SD = 1.70).

Most hospitals (64.6%) did not have areas specifically designated for palliative care patients. Only 3 hospitals (0.6%) had a ward allocated for palliative care, 28 hospitals (5.2%) had a palliative care zone, and 161 hospitals (29.7%) had dedicated beds for palliative care patients. Only 114 hospitals (21.03%) reported that they had conducted research on palliative care. Regarding the process of care, the average time spent by medical staff per patient was 22.19 min (SD = 13.0) for doctors and 40.14 min (SD = 15.30) for nurses. Table 1 presents the descriptive statistics of the participating hospitals.

**Table 1:** Characteristics of the study sample (*n* = 542)

**Variables**	**Frequency (%)**	**Mean**	**SD**
Hospital Size			
Number of beds		103.15	147.12
Sufficiency of Medical Personnel			
Doctor-to-Palliative Patient Ratio		0.38	0.42
Nurse-to-Palliative Patient Ratio		1.61	2.11
Staff Competency			
Palliative care-trained doctors in facility (Yes)	56 (10.33)		
(Number of trained doctors in facility)		0.15	0.57
Palliative care-trained nurses in facility (Yes)	274 (50.55)		
(Number of trained nurses in facility)		0.81	1.13
Types of Multidisciplinary Team Members			
Social Workers in multidisciplinary team (Yes)	63 (11.62)		
(Number of social workers in team)		0.13	0.36
Psychologists in multidisciplinary team (Yes)	182 (33.58)		
(Number of psychologists in team)		0.35	0.50
Variety of Professions in Multidisciplinary Team		5.13	1.55
Variety of Medical Equipment in Facility		3.24	0.77
Syringe drivers (Yes)	241 (44.46)		
Infusion pumps (Yes)	346 (63.84)		
Oxygen concentrators (Yes)	369 (68.08)		
Airbeds (Yes)	424 (78.23)		
Variety of Relaxation Equipment in Facility		3.48	1.70
Prayer room (Islam) (Yes)	60 (11.07)		
Video (Yes)	93 (17.16)		
Tabernacle (Yes)	123 (22.69)		
Radio (Yes)	232 (42.80)		
TV (Yes)	241 (44.46)		
Magazine/Newspaper/Cartoon (Yes)	256 (47.23)		
Dhamma cassette (Yes)	394 (72.69)		
Dhamma book (Yes)	485 (89.48)		
Availability of Medicine in Facility		4.35	1.81
Oxycodone (Yes)	6 (1.11)		
Methadone (Yes)	42 (7.75)		
Codeine (Yes)	78 (14.39)		
Fentanyl patch (Yes)	83 (15.31)		
Kapanol (Yes)	95 (17.53)		
Fentanyl injection (Yes)	121 (22.32)		
Morphine immediate release (Yes)	200 (36.90)		
Morphine syrup (Yes)	335 (61.81)		
MST (Yes)	341 (62.92)		
Morphine injection (Yes)	523 (96.49)		
Tramadol (Yes)	531 (97.97)		
Research in Palliative Care (Yes)	114 (21.03)		
Presence of Designated Area for Palliative Patients			
Non-specific area	350 (64.58)		
Palliative beds	161 (29.70)		
Palliative zone (apart from ward)	28 (5.17)		
Palliative ward	3 (0.55)		
Time Spent per patient (minutes)			
Doctor		22.19	13.00
Nurse		40.14	15.30

### Reliability and validity of the latent variables

Table 2 presents the results of the reliability and validity analyses of the palliative care process latent variables. The Cronbach’s α values for the variables *response to physical and psychological needs* and *effective communication* were found to exceed 0.7 (Cronbach’s α = 0.88 and 0.83, respectively), indicating good internal consistency [29, 30]. However, the variable *respect for patient* had a lower internal consistency (Cronbach’s α = 0.45), and the elimination of the indicator *living will* resulted in acceptable internal consistency (Cronbach’s α = 0.71). The results of the confirmatory factor analyses revealed acceptable fit indices for all three latent variables, indicating the validity of the constructs.

**Table 2:** Reliability and validity of palliative care process latent variables (*n* = 542)

**Latent Variables**	**Internal consistency Cronbach’s α**	**Corrected Item-Total Correlation**	**Cronbach’s α of Domain if Item Deleted**	**CFA Factor loading**
Response to patient’s needs	0.88			
Pain management		0.59	0.88	.64***
Delirium management		0.72	0.85	.71***
Depression management		0.75	0.85	.75***
Spiritual care management		0.78	0.84	.88***
Bereavement care management		0.74	0.85	.83***
Effective Communication	0.83			
Compliance to breaking bad news (BBN) guidelines		0.64	0.79	.72***
Compliance to Advance care planning (ACP) guidelines		0.68	0.78	.78***
BBN by trained staff		0.66	0.79	.72***
ACP by trained staff		0.65	0.79	.75***
Respect to patient dignity	0.45			
Decision-making		0.40	0.25	.77***
Living will		0.19	0.71^a^	.23***
Place of death		0.37	0.24	.72***
All 11 items	0.90			

Model fit index of CFA for the response to physical and psychological needs: χ^2^/df = 1.713 (*p*-value = 0.144), GFI = 0.995, AGFI = 0.982, CFI = 0.998, RMSEA = 0.036, and SRMR = 0.0120

Model fit index of CFA for effective communication: χ^2^/df = 0.419 (*p*-value = 0.518), GFI = 1.000, AGFI = 0.996, CFI = 1.000, RMSEA = 0.000, and SRMR = 0.0051

Model fit index of CFA for the Respect to patient: -

*** *p* < .01

^a^removed from the model

### Structural equation modelling

**Fig. 1:**
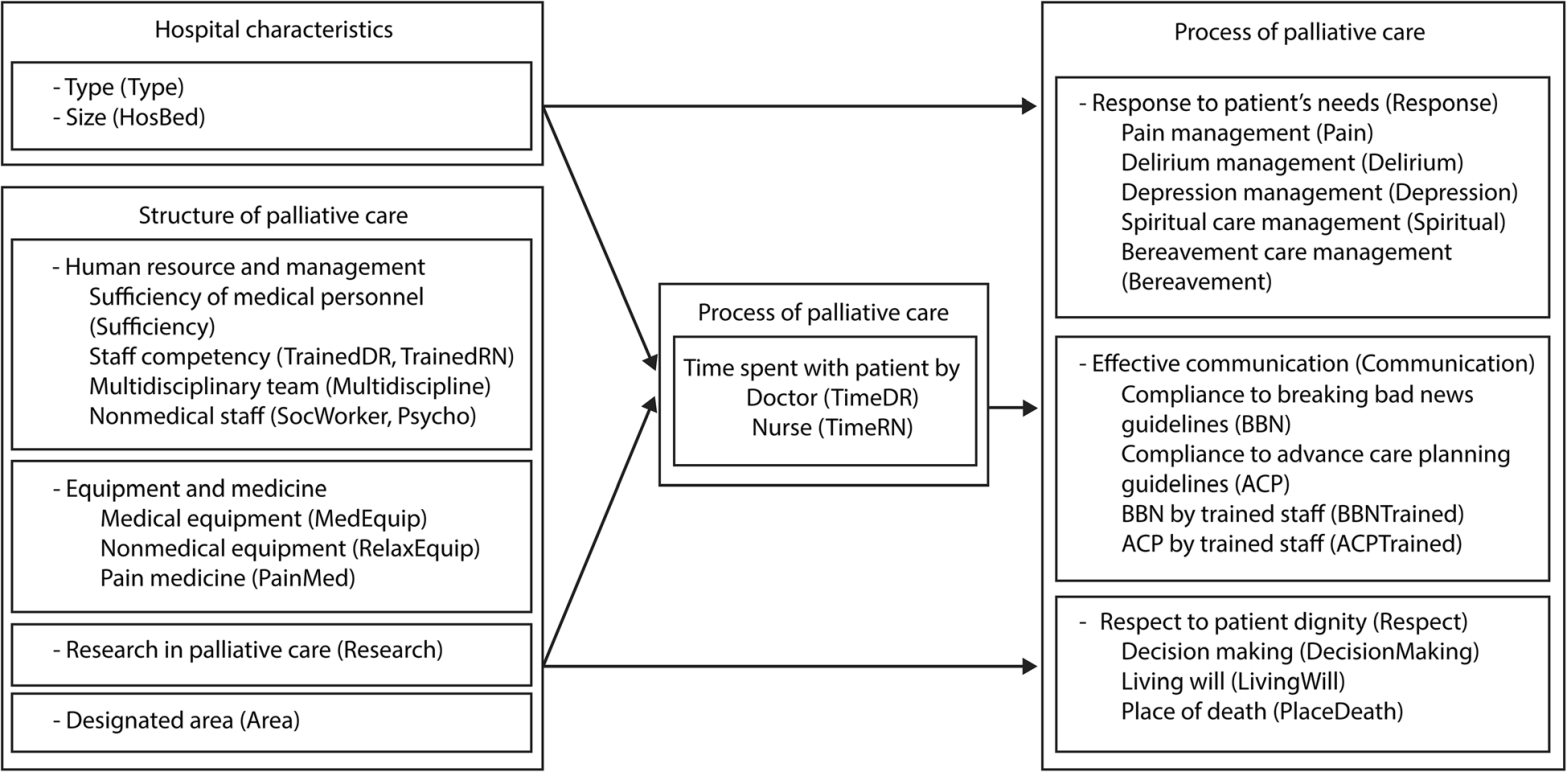
An analytical framework of the hypothesized relationships between the structure and process of palliative care (Name of variables in the SEM model)

SEM was conducted to simultaneously examine the relationship between a hospital’s palliative care structure and the process of care, as hypothesized in Donabedian’s framework [31]. The hospital structure variables, including *type of hospital* [Type], *size* [HosBed], *sufficiency of medical staff* [Sufficiency], *staff competency* [TrainedDR, TrainedRN], *availability of a multidisciplinary team* [Multidiscipline], *number of social workers* [SocWorker], *number of psychologists* [Psycho], *variety of medical equipment* [MedEquip], *variety of relaxation equipment* [RelaxEquip], *availability of pain medicine* [PainMed], *research on palliative care* [Research], and *presence of designated area for palliative patients* [Area], were hypothesized to have a direct influence on the process of care variables, which are *response to physical and psychological needs* [Response], *effective communication* [Communication], and *respect for patient’s dignity* [Respect] and an indirect influence through *time spent by doctors* [TimeDR] and *time spent by nurses* [TimeRN]. The model fit statistics for the hypothesized model were poor (χ^2^/df = 10.784 (*p* = 0.000), GFI = 0.696, AGFI = 0.573, CFI = 0.545, RMSEA = 0.134, and SRMR = 0.165). Figure 1 shows an analytical framework of the hypothesized relationships between the structure and process of palliative care.

**Fig. 2:**
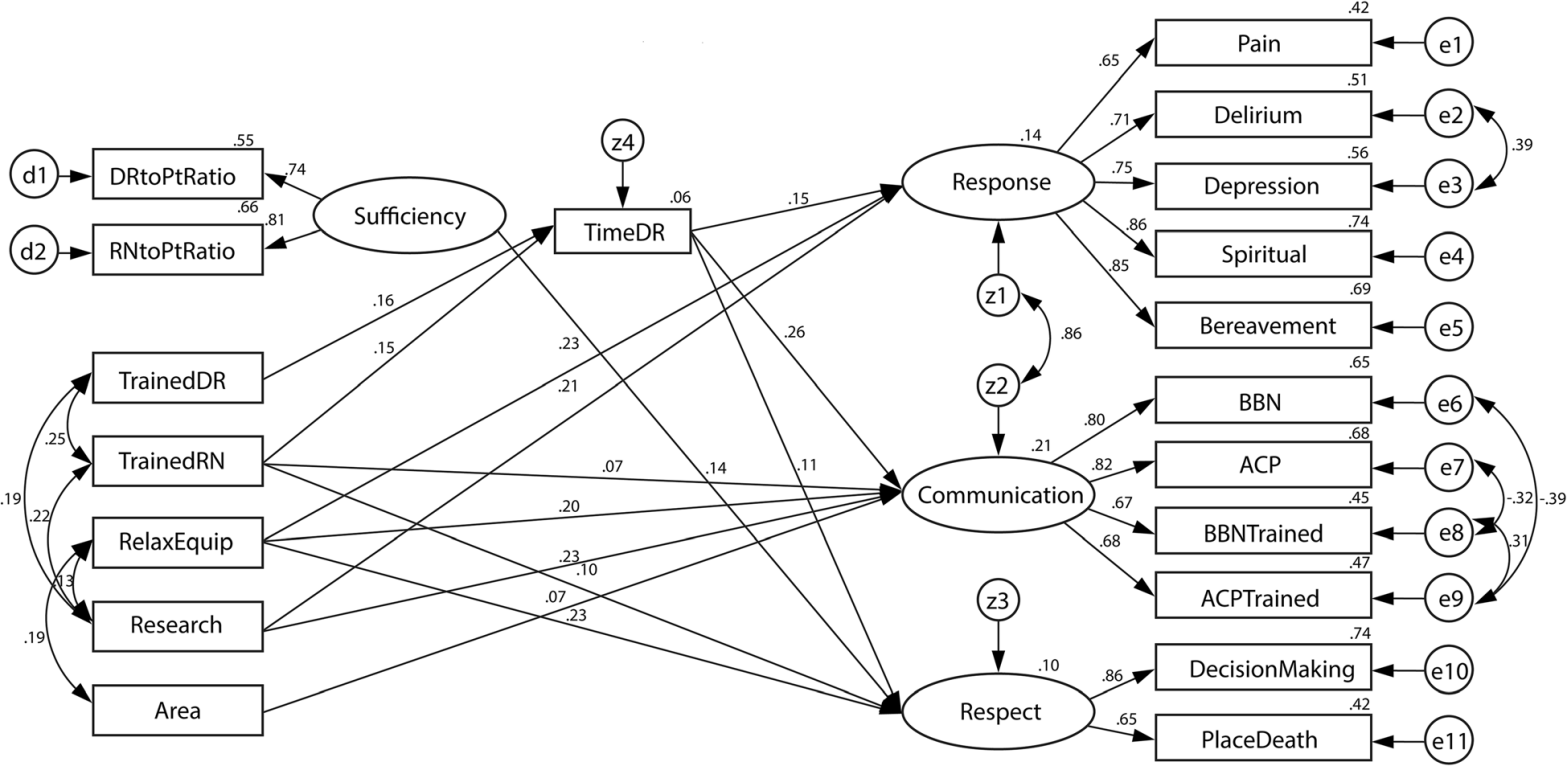
A revised SEM model for palliative care quality

The model was revised, with insignificant paths removed and measurement errors allowed to be correlated. Figure 2 shows the revised SEM model with only the significant associations among the variables. Variety of relaxation equipment [RelaxEquip] (γ = 0.23, *p* < 0.001) and research on palliative care [Research] (γ = 0.21, *p* < 0.001) had significantly positive associations with response to physical and psychological needs [Response]. The number of nurses trained in palliative care [TrainedRN] (γ = 0.07, *p* = 0.013), variety of relaxation equipment [RelaxEquip] (γ = 0.20, *p* < 0.001), research on palliative care [Research] (γ = 0.23, *p* < 0.001), and presence of designated areas for palliative patients [Area] (γ = 0.07, *p* = 0.013) were significantly positively associated with effective communication [Communication]. Sufficiency of medical staff [Sufficiency] (γ = 0.14, *p* = 0.014), number of nurses trained in palliative care [TrainedRN] (γ = 0.10, *p* < 0.001) and variety of relaxation equipment [RelaxEquip] (γ = 0.23, *p* < 0.001) were significantly positively associated with respect for patient [Respect]. Furthermore, the two variables of staff competency, number of doctors trained in palliative care [TrainedDR] (γ = 0.16, *p* < 0.001) and number of nurses trained in palliative care [TrainedRN] (γ = 0.15, *p* < 0.001), had indirect influences on the three processes concerning good death variables through the variable doctor’s time spent with patients [TimeDR], which was significantly associated with Response (β = 0.15, *p* < 0.001), Communication (β = 0.26, *p* < 0.001) and Respect (β = 0.11, *p* = 0.026). A total of 14, 21 and 10% of the variation in the variables Response, Communication and Respect was explained by this model, respectively. The model fit statistics show that the model fits the data well (χ^2^/df = 2.705, GFI = 0.937, AGFI = 0.910, CFI = 0.940, RMSEA = 0.056, and SRMR = 0.0807). Table 3 shows the standardized and unstandardized estimates of the associations between the variables in the SEM model.

**Table 3:** Parameter estimates for the revised model

**Variables**			**Standardized Regression Coefficient**	**Unstandardized Regression Coefficient**	**S.E.**	**C.R.**
TimeDR	<---	TrainedDR	0.16	3.60	0.98	3.66***
TimeDR	<---	TrainedRN	0.15	1.76	0.49	3.56***
Response	<---	RelaxEquip	0.23	3.57	0.70	5.07***
Response	<---	Research	0.21	13.83	2.94	4.71***
Response	<---	TimeDR	0.15	0.31	0.09	3.48***
Communication	<---	TrainedRN	0.07	2.28	0.92	2.48**
Communication	<---	Research	0.23	20.48	3.76	5.44***
Communication	<---	Area	0.07	2.74	1.10	2.48**
Communication	<---	RelaxEquip	0.20	4.15	0.89	4.65***
Communication	<---	TimeDR	0.26	0.70	0.12	6.01***
Respect	<---	RelaxEquip	0.23	2.31	0.49	4.75***
Respect	<---	Sufficiency	0.14	7.97	3.24	2.46**
Respect	<---	TimeDR	0.11	0.14	0.06	2.23**
Respect	<---	TrainedRN	0.10	1.61	0.73	2.19**

** *p* < 0.05 *** *p* < 0.01

## Discussion

To our knowledge, this is the first nationwide study to explore the overall situation of facility-based palliative care in Thailand. Specifically, this study investigates how palliative care provision systems are arranged in hospitals and whether they affect the process of care, which can enhance the quality of life of patients.

The results of this study indicate that the sufficiency and competency of the medical staff and hospitals’ nonmedical structure regarding palliative care are associated with the process of care, including the response to physical and psychological needs, effective communication and respect for patients.

Staffing in palliative care has been found to be associated with better quality of care. Hospitals with more medical staff can provide consistent care and are more responsive to palliative care patients’ needs [32, 33]. In this study, doctors and nurses were the key staff members that contributed to the palliative care process. In addition to sufficiency, the competency of palliative care staff is crucial to the quality of palliative care provision. The number of doctors and the number of nurses who had taken an intensive palliative care course were found to have a significant impact on the three aspects of the care process either directly or indirectly through the amount of time that the doctor spent with a patient. Spending more time with patients enables doctors to build a curative relationship and foster rapport, which demonstrates empathy and allows patients to express their psychosocial concerns [34]. This finding is supported by recent studies [35–39] showing that doctors spending more time discussing information about disease or treatment guidelines in prognostic conversations allows patients to gain a greater understanding of their illness and treatment options.

Although the time nurses spend with patients is not significantly associated with the process of care, palliative care nurses generally spend more time with patients and their families than other health professionals do [40, 41]. In addition, the interactions between nurses and patients are more informal than those between patients and doctors. Palliative care nurses play the main roles in individualized care [42], detailed communication about treatment [43] and facilitation of patients’ decision making regarding their end of life. The direct influence of the number of palliative care-trained nurses on effective communication and respect for patients implies the importance of the nonclinical functions of nurses in care practices and the need to strengthen these in the context of palliative care.

These findings emphasize the importance of knowledge and skills specific to palliative care that improve the competency of medical staff in responding to patient needs, leading to the initiation of end-of-life care discussions and planning with patients and families. However, the descriptive statistics from this study show that only 10% of participating hospitals had at least one doctor and 50% had at least one nurse who had received at least 1 month of training through an intensive course on palliative care. This finding confirms that palliative care training for both doctors and nurses through existing curricula in Thailand is still not adequate, as previously reported [1, 6]. Therefore, promoting intensive training among medical staff should be considered a priority for the improvement of palliative care quality in this country. Given the differences in the specific functions of doctors and nurses, palliative care courses could be designed to target different medical professions and optimize the knowledge and skills obtained.

Although we found clear differences across hospitals with different levels of capacity in some structural components, namely, the variety of medical professions on the multidisciplinary team, availability of medical equipment and availability of medication for palliative care patients, the associations between these medically related components and the process of care were not significant. On the other hand, nonmedical components such as the availability of relaxation equipment, allocation of specific areas for palliative care patients and research on palliative care appear to be related to the quality of the care process. These managerial structural variables could imply what a hospital is capable of and give value to palliative care provision.

In the palliative care context, relaxation equipment such as music and prayer recordings have been found to reduce pain while promoting relaxation and relieving anxiety and depression in patients [44–46]. In Thai society, which is predominantly Buddhist, patients and families facing the end-of-life stage of disease are likely to listen to Dharmic recordings and read Dharmic books. It is believed that patients and their families can learn to accept, prepare and plan as they make the decision to discontinue invasive treatments and unnecessary life-prolonging procedures. Thai society remains largely religious; hence, religious activities for followers of different religions, such as prayers, Quran reading, and anointing, are commonly observed in hospitals in the presence of clergymen or Buddhist monks [47]. Religious patients can thereby cope with the physical, psychological and spiritual effects of the final stage of life [48, 49]. Having a variety of relaxation equipment may indicate that hospitals’ preparedness extends beyond physical and psychosocial care to spiritual care and respect for patients’ dignity. This explains the relationship between the variety of relaxation equipment and all three aspects of the process of palliative care.

The allocation of specific areas to serve palliative care patients can lead to a better process of care, particularly with respect to the communication of sensitive issues [50]. Thai medical practitioners are expected to follow the guidelines for palliative care communication, particularly when delivering bad news and discussing care planning with patients and family members [11, 51]. These are sensitive issues that might have psychological impacts on patients whose condition is clinically unstable or made complex by the symptoms of disease. Being in an organized, private zone with whom patients are familiar can have positive effects on patient quality of life [52]. The significant association between having an area specifically designated for palliative care and effective communication, in one way, suggests that the challenges in palliative care communication can be overcome if hospitals take into consideration the necessity of physical space for patients and their family caregivers.

Research on palliative care might lead to practice guidelines, quality indicators or specific tools for better care performance [53, 54]. Hospitals that have conducted research on palliative care were found to have a better quality of care process in terms of response to patients’ needs and effective communication. As mentioned earlier, most hospitals in Thailand reported following guidelines developed by their specific facility [12], which implies a wider interest in improving the quality of palliative care at the facility level. However, the number of hospitals that have experience conducting research, as observed in this study, is quite limited. Among the palliative care studies that have been performed so far, most are descriptive, and there is little intervention research related to the promotion of quality of life among patients and their families [1]. Although this study was not an intervention study, it confirms the significance of research at the facility level for the improvement of the palliative care process.

In this study setting, we cannot capture the differences in the quality of the palliative care process between hospitals with higher and lower medical capacity. This might be due to the nature of palliative care, which relies more on psychosocial-spiritual aspects of care; thus, patients’ physical condition can be managed well even with less advanced medical equipment and medication. It should be noted that both financial and technical support are vital for the investment in improving human resources, medical and nonmedical equipment, the physical environment and other related care resources. As such, hospitals’ managerial philosophy and organizational culture contribute substantially in this regard. In future studies, hospitals’ structural characteristics, particularly the managerial and cultural aspects of healthcare organizations, should be included to systematically explain palliative care quality management in greater detail.

This study employs Donabedian’s S-P-O framework by assuming that the quality of the structure and process of palliative care leads to the ultimate outcome of good death. However, no actual outcome data were directly collected in this study, for two main reasons. First, palliative care outcomes have been measured in different ways, mostly in terms of satisfaction with the care provided and not in terms of good death and the dying process. There is no standard quantification of palliative care outcomes that we can observe in the study setting. Second, this is a cross-sectional study, so the ability to infer causal links from structure to process to outcome, according to the theory, is limited. However, the structure and process measures of, for example, the availability and competency of human resources, the availability of pain killers, psychosocial support for patients and families and support for shared decision making are among the indicators in the Quality of Good Death Index [55]. We believe that the study results indicate what structural components are essential for better care processes and how palliative care provision should be designed at the facility level.

Taking an organization as a unit of analysis generally requires objective measurement of organizational-level variables. Thailand is in an active phase of developing guidelines and quality standards for palliative care [9, 56]; however, as mentioned earlier, hospitals apply different performance indicators. Therefore, we used objectively observed variables that can typically be obtained from any hospital to construct the measurement models for the three aspects of care. Accordingly, these data allowed us to conduct a quantitative evaluation of the overall situation and a comparison of palliative care provision performance across medical facilities nationwide despite a standard quality measurement not yet being available.

This study has some limitations that need to be addressed. First, the study setting is limited to institutional care, which mostly involves patients approaching the end of life. Even though medically related structural components and staff specialization were not found to be significant predictors of the palliative care process in this study, we cannot conclude that they are not important. Palliative patients in different stages of disease progression may have different needs regarding the structure and process of care. The specific needs of patients in different stages of disease progression could be further explored. In addition, the provision of palliative care extends beyond institutional care. Patients may prefer to receive care at home or in the community. Community-based palliative care integrates specialized care with the local health system while enhancing family and community involvement to ensure continuity of care and maintain patients’ quality of life and dignity. Therefore, the quality of palliative care cannot be assessed only at medical facilities but must encompass the entire care system.

Second, this study uses objective measurement based on data that any hospital can provide; however, it fully relies on primary data collected using questionnaires self-administered by hospital representatives. Some information may be estimated and thus may be affected by response bias. Facility- and national-level databases that include standard indicators for palliative care should be developed to facilitate potential research for quality improvement. Third, the structure of care is operationalized using directly observable components such as count, variety and the presence of human and physical resources, but managerial style, culture, leadership, policies and other organization-level factors that might affect how palliative care is managed and provided are not included. This limits the interpretation of the study findings in terms of how a medical facility can provide better quality of palliative care. Future studies on the role of the structural characteristics of hospitals in determining the quality of palliative care are encouraged.

## Conclusion

This is the first nationwide study to explore the associations between the structure and process of palliative care in Thai hospitals. The study revealed that the sufficiency and competency of palliative care doctors and nurses, together with the presence of nonmedical equipment, the allocation of physical space, and the presence of research related to palliative care, are associated with the process of care, which entails response to patients’ physical and psychological needs, effective communication and respect for patients’ dignity. Financial and technical support at the facility and national levels for investment in the structural components of care is essential to improve the quality of palliative care and patients’ quality of life.

## Data Availability

The dataset used in the current study is not publicly available given the conditions stated in the informed consent form to protect the identity of the participants. However, the dataset is available from the corresponding author on reasonable request.

## References

[1] Nilmanat K (2016). Palliative care in Thailand: development and challenges. Can Oncol Nurs J.

[2] Strategy and Planning Division. Ministry of Public Health, Thailand; 2019.

[3] Clark D, Baur N, Clelland D, Garralda E, López-Fidalgo J, Connor S, Centeno C (2020). Mapping levels of palliative care development in 198 countries: the situation in 2017. J Pain Symptom Manag.

[4] Goh CR, Lee SY (2018). Education in pain and palliative care in the low-and middle-income countries of the Asia Pacific region. Pain.

[5] Vallath N, Rajagopal MR, Perera S, Khan F, Paudel BD, Tisocki K (2018). Access to pain relief and essential opioids in the WHO South-East Asia region: challenges in implementing drug reforms. WHO South East Asia J Public Health.

[6] Nilmanat K, Udchumpisai M, Potjamanpong, NiyomThai N (2019). Continuing hospital-to-home palliative care: a southern Thai context. TJNC.

[7] Noparit P, Promdee A (2020). Development of clinical nursing practice guideline for palliative care in patients with end stage renal disease Mukdahan Hospital. MKHJ.

[8] Siriloadjanamanee K, Soivong P, Phornphibul P (2019). Breaking bad news in palliative care: integrative review. Nurs J.

[9] Wonghongkul T, Mesukko J, Charuwatcharapaniskul U, Gomuthbuth P (2016). Development of palliative care indicators. Int J Evid Based Healthc.

[10] Kitreerawutiwong N, Mekrungrengwong S, Keeratisiroj O (2018). The development of the community-based palliative care model in a district health system, Phitsanulok Province, Thailand. Indian J Palliat Care.

[11] Mesukko J, Turale S, Jintrawet U, Niyomkar S (2020). Palliative care guidelines for physicians and nurses caring for children and their families in the pediatric intensive care units: a participatory action research study. Pac Rim Int J Nurs Res.

[12] Srirattanaban J, Chatkaew P, Srirattanaban K, Nhamkiatphaisan S, Manausvanich P, Rattananupong T (2018). The study of health system services for hospice care provision in Thailand.

[13] Bureau of Inspection. Summary of Ministry of Public Health inspection year 2020 first round. [Internet] Nonthaburi: Ministry of Public Health Bureau of Inspection; 2020 [updated 2020 Jun; cited 2020 Oct 12]. Available from: http://strategy.skto.moph.go.th/documents/post/%E0%B8%AA%E0%B8%A3%E0%B8%B8%E0%B8%9B%E0%B8%95%E0%B8%A3%E0%B8%A7%E0%B8%88%E0%B8%A3%E0%B8%B2%E0%B8%8A%E0%B8%81%E0%B8%B2%E0%B8%A31-2563.pdf.

[14] Strategy and Planning Division. Roadmap, programs and indicators under the Ministry of Public Health government action plan fiscal year 2020. [Internet] Nonthaburi: Ministry of Public Health; 2020 [cited 2020 Oct 12]. Available from: https://guides.library.uq.edu.au/referencing/vancouver/government#s-lg-box-18992039.

[15] Hui D, Kim YJ, Park JC, Zhang Y, Strasser F, Cherny N (2015). Integration of oncology and palliative care: a systematic review. Oncologist.

[16] Albers G, Froggatt K, Van den Block L, Gambassi G, Berghe PV, Pautex S, Van Den Noortgate N (2016). A qualitative exploration of the collaborative working between palliative care and geriatric medicine: barriers and facilitators from a European perspective. BMC Palliat Care.

[17] Aoyama M, Morita T, Kizawa Y, Tsuneto S, Shima Y, Miyashita M (2017). The Japan HOspice and Palliative Care Evaluation Study 3: study design, characteristics of participants and participating institutions, and response rates. Am J Hosp Palliat Med.

[18] Pfaff K, Markaki A (2017). Compassionate collaborative care: an integrative review of quality indicators in end-of-life care. BMC Palliat Care.

[19] Buja A, Rivera M, Baldo V, Soattin M, Rizzolo Y, Zamengo G, et al. Palliative care quality measures: an exploratory study. BMJ Support Palliat Care. 2019. 10.1136/bmjspcare-2018-001679. 10.1136/bmjspcare-2018-001679 30765387

[20] Hasson F, Nicholson E, Muldrew D, Bamidele O, Payne S, McIlfatrick S (2020). International palliative care research priorities: a systematic review. BMC Palliat Care.

[21] Donabedian A (2003). An introduction to quality assurance in health care.

[22] Hjermstad MJ, Gibbins J, Haugen DF, Caraceni A, Loge JH, Kaasa S, EPCRC, European Palliative Care Research Collaborative (2008). Pain assessment tools in palliative care: an urgent need for consensus. Palliat Med.

[23] Granda-Cameron C, Houldin A (2012). Concept analysis of good death in terminally ill patients. Am J Hosp Palliat Med.

[24] Doorenbos AZ, Juntasopeepun P, Eaton LH, Rue T, Hong E, Coenen A (2013). Palliative care nursing interventions in Thailand. J Transcult Nurs.

[25] Kamal AH, Gradison M, Maguire JM, Taylor D, Abernethy AP (2014). Quality measures for palliative care in patients with cancer: a systematic review. J Oncol Pract.

[26] Good MJD, Gadmer NM, Ruopp P, Lakoma M, Sullivan AM, Redinbaugh E (2004). Narrative nuances on good and bad deaths: internists’ tales from high-technology work places. Soc Sci Med.

[27] Thompson G, McClement S, Daeninck P (2006). Nurses’ perceptions of quality end-of-life care on an acute medical ward. J Adv Nurs.

[28] Hu LT, Bentler PM (1999). Cutoff criteria for fit indexes in covariance structure analysis: conventional criteria versus new alternatives. Struct Equ Model Multidiscip J.

[29] George D, Mallery P (2003). SPSS for windows step by step: a simple guide and reference 11.0 update.

[30] Hair JF Jr, Black WC, Babin BJ, Anderson RE. Multivariate data analysis (7th ed). Harlow: Pearson New International Edition; 2014.

[31] Donabedian A (1966). Evaluating the quality of medical care. Milbank Mem Fund Q.

[32] Wentlandt K, Seccareccia D, Kevork N, Workentin K, Blacker S, Grossman D, Zimmermann C (2016). Quality of care and satisfaction with care on palliative care units. J Pain Symptom Manag.

[33] Kim SN, Choi SO, Shin SH, Ryu JS, Baik JW (2017). Development of a community-based palliative care model for advance cancer patients in public health centers in Busan, Korea. Cancer Res Treat.

[34] Braddock CH, Snyder L (2005). The doctor will see you shortly. J Gen Intern Med.

[35] Moore PM, Rivera S, Bravo-Soto GA, Olivares C, Lawrie TA. Communication skills training for healthcare professionals working with people who have cancer. Cochrane Database Syst Rev. 2018;(7). 10.1002/14651858.CD003751.pub4. 10.1002/14651858.CD003751.pub4 PMC6513291 30039853

[36] Back AL, Fromme EK, Meier DE (2019). Training clinicians with communication skills needed to match medical treatments to patient values. J Am Geriatr Soc.

[37] Turrillas P, Teixeira MJ, Maddocks M (2019). A systematic review of training in symptom management in palliative care within postgraduate medical curriculums. J Pain Symptom Manag.

[38] Anderson RJ, Stone PC, Low JT, Bloch S. Managing uncertainty and references to time in prognostic conversations with family members at the end of life: a conversation analytic study. Palliat Med. 2020. 10.1177/0269216320910934. 10.1177/0269216320910934 PMC7336362 32233831

[39] Nevin M, Hynes G, Smith V (2020). Healthcare providers’ views and experiences of non-specialist palliative care in hospitals: a qualitative systematic review and thematic synthesis. Palliat Med.

[40] Ferrell B, Malloy P, Virani R (2015). The end of life nursing education nursing consortium project. Ann Palliat Med.

[41] Sekse RJT, Hunskår I, Ellingsen S (2018). The nurse’s role in palliative care: a qualitative meta-synthesis. J Clin Nurs.

[42] Anderson RJ, Bloch S, Armstrong M, Stone PC, Low JT (2019). Communication between healthcare professionals and relatives of patients approaching the end-of-life: a systematic review of qualitative evidence. Palliat Med.

[43] Warnock C, Buchanan J, Tod AM (2017). The difficulties experienced by nurses and healthcare staff involved in the process of breaking bad news. J Adv Nurs.

[44] Ahluwalia SC, Chen C, Raaen L, Motala A, Walling AM, Chamberlin M (2018). A systematic review in support of the national consensus project clinical practice guidelines for quality palliative care. J Pain Symptom Manag.

[45] Yeh IM, Zhang H (2018). New media, part six:“patient-centered” mobile apps for meditation and stress reduction. J Palliat Med.

[46] Peng CS, Baxter K, Lally KM (2019). Music intervention as a tool in improving patient experience in palliative care. Am J Hosp Palliat Med.

[47] Kongsuwan W, Chaipetch O, Matchim Y (2012). Thai Buddhist families’ perspective of a peaceful death in ICUs. Nurs Crit Care.

[48] Matetanonto M (2005). Four religions in the end-of-life care.

[49] Choudry M, Latif A, Warburton KG (2018). An overview of the spiritual importances of end-of-life care among the five major faiths of the United Kingdom. Clin Med.

[50] Street AF, Love A (2005). Dimensions of privacy in palliative care: views of health professionals. Soc Sci Med.

[51] Boonnun J, Chayaput P (2010). Nurse’s role in unfavorable information “breaking bad news” communication to cancer patients.

[52] Slatyer S, Pienaar C, Williams AM, Proctor K, Hewitt L (2015). Finding privacy from a public death: a qualitative exploration of how a dedicated space for end-of-life care in an acute hospital impacts on dying patients and their families. J Clin Nurs.

[53] Sleeman KE, Koffman J, Bristowe K, Rumble C, Burman R, Leonard S, et al. ‘It doesn’t do the care for you’: a qualitative study of health care professionals’ perceptions of the benefits and harms of integrated care pathways for end of life care. BMJ Open. 2015;5(9). 10.1136/bmjopen-2015-008242. 10.1136/bmjopen-2015-008242 PMC4577969 26369795

[54] Higginson IJ (2016). Research challenges in palliative and end of life care. BMJ Support Palliat Care.

[55] The Economist Intelligence Unit. The 2015 Quality of Death Index: Ranking palliative care across the world. [Internet] London: Lien Foundation; 2015 [updated 2015 Oct; cited 2017 Apr 10]. Available from: https://eiuperspectives.economist.com/sites/default/files/2015%20EIU%20Quality%20of%20Death%20Index%20Oct%2029%20FINAL_0.pdf.

[56] Karunruk Palliative Care Center. Quality standards for palliative care. Khon Kaen: Klungnanavitthaya Press; 2018.

